# Activation of PLCβ1 enhances endocannabinoid mobilization to restore hippocampal spike-timing-dependent potentiation and contextual fear memory impaired by Alzheimer’s amyloidosis

**DOI:** 10.1186/s13195-021-00901-9

**Published:** 2021-10-08

**Authors:** Jaedong Lee, Jeehyun Kwag

**Affiliations:** Department of Brain and Cognitive Engineering, Korea University, 145 Anam-ro, Seongbuk-gu, Seoul, South Korea

**Keywords:** Alzheimer’s disease, Hippocampus, Endocannabinoid mobilization, PLCβ1, Long-term potentiation, Contextual fear memory

## Abstract

**Background:**

Accumulation of amyloid beta oligomers (AβO) in Alzheimer’s disease (AD) impairs hippocampal long-term potentiation (LTP), leading to memory deficits. Thus, identifying the molecular targets of AβO involved in LTP inhibition is critical for developing therapeutics for AD. Endocannabinoid (eCB) synthesis and release, a process collectively called eCB mobilization by hippocampal CA1 pyramidal cells, is known to facilitate LTP induction. eCB can be mobilized either by postsynaptic depolarization in an intracellular Ca^2+^ concentration ([Ca^2+^]_i_)-dependent pathway or by group 1 metabotropic glutamate receptor (mGluR) activation in a phospholipase Cβ (PLCβ)-dependent pathway. Moreover, group 1 mGluR activation during postsynaptic depolarization, which is likely to occur in vivo during memory processing, can cause synergistic enhancement of eCB (S-eCB) mobilization in a PLCβ-dependent pathway. Although AβO has been shown to disrupt [Ca^2+^]_i_-dependent eCB mobilization, the effect of AβO on PLCβ-dependent S-eCB mobilization and its association with LTP and hippocampus-dependent memory impairments in AD is unknown.

**Methods:**

We used in vitro whole-cell patch-clamp recordings and western blot analyses to investigate the effect of AβO on PLCβ protein levels, PLCβ-dependent S-eCB mobilization, and spike-timing-dependent potentiation (tLTP) in AβO-treated rat hippocampal slices in vitro. In addition, we assessed the relationship between PLCβ protein levels and hippocampus-dependent memory impairment by performing a contextual fear memory task in vivo in the 5XFAD mouse model of AD.

**Results:**

We found that AβO treatment in rat hippocampal slices in vitro decreased hippocampal PLCβ1 protein levels and disrupted S-eCB mobilization, as measured by western blot analysis and in vitro whole-cell patch-clamp recordings. This consequently led to the impairment of NMDA receptor (NMDAR)-mediated tLTP at CA3-CA1 excitatory synapses in AβO-treated rat hippocampal slices in vitro. Application of the PLCβ activator, m-3M3FBS, in rat hippocampal slices reinstated PLCβ1 protein levels to fully restore S-eCB mobilization and NMDAR-mediated tLTP. In addition, direct hippocampal injection of m-3M3FBS in 5XFAD mice reinstated PLCβ1 protein levels to those observed in wild type control mice and fully restored hippocampus-dependent contextual fear memory in vivo in 5XFAD mice.

**Conclusion:**

We suggest that these results might be the consequence of memory impairment in AD by disrupting S-eCB mobilization. Therefore, we propose that PLCβ-dependent S-eCB mobilization could provide a new therapeutic strategy for treating memory deficits in AD.

## Background

Alzheimer’s disease (AD) is a neurodegenerative disorder mainly characterized by a progressive memory impairment and cognitive decline [1–3]. It has been proposed that abnormal accumulation of soluble amyloid beta oligomers (AβO), a proteolytic derivative of the amyloid precursor protein, could be one of the causes underlying the neurobehavioral alterations in AD [3–5]. AβO has deleterious effects on neurons and synapses, leading to synaptic transmission dysfunction [3] ﻿, dendritic spine loss [6, 7], and neural death [8, 9]. Most importantly, several studies suggest that AβO impairs long-term potentiation (LTP) at hippocampal excitatory synapses [4, 10, 11], the most probable synaptic mechanism underlying memory [12, 13]. Thus, understanding which molecular and synaptic mechanisms underlying LTP are targeted by AβO will be critical in developing therapeutic targets for restoring LTP and, consequently, the memory deficits observed in AD.

Among the many molecular and synaptic mechanisms involved in the induction of LTP, endocannabinoids (eCBs) such as 2-arachidonoylglycerol (2-AG) are known to directly control the induction of hippocampal NMDA receptor (NMDAR)-mediated LTP [14, 15]. eCB is synthesized and released from postsynaptic hippocampal CA1 pyramidal cells (PCs) through a process collectively called eCB mobilization [16]. eCB acts as a retrograde messenger by binding to the presynaptic cannabinoid type 1 receptor (CB1R) to decrease the release of presynaptic neurotransmitters [17, 18] such as gamma-aminobutyric acid (GABA) [19, 20] onto CA1 PCs. eCB mobilization facilitates the induction of NMDAR-mediated LTP at hippocampal CA3-CA1 synapses [14, 15, 21, 22], while blockade of CB1R impairs the induction of NMDAR-mediated LTP [14, 15, 22]. This indicates the critical involvement of eCB mobilization in LTP induction. Interestingly, hippocampal CB1R density is substantially decreased in patients with AD [23] and in the amyloid precursor protein (APP)/presenilin-1 (PSEN1) mouse model of AD [24]. It has been shown that both the application of CB1R agonists [25–27] and also the inactivation of the activity of monoacylglycerol lipase (MAGL), the enzyme that hydrolyzes 2-AG, result in an increase of 2-AG levels and restore LTP in a mouse model of AD [28], suggesting critical involvement of the eCB system in hippocampal LTP induction. However, the stage at which the eCB mobilization process is disrupted by AβO to impair LTP is unknown.

To identify the targets of AβO in the hippocampal eCB system, it is important to understand how eCB is mobilized from CA1 PCs. eCB can be mobilized either by postsynaptic depolarization of CA1 PCs [19, 29] or by the activation of group 1 metabotropic glutamate receptors (mGluRs) [30, 31]. Prolonged (5–10 s) postsynaptic depolarization of CA1 PCs opens voltage-gated calcium (Ca^2+^) channels to increase intracellular Ca^2+^ concentration ([Ca^2+^]_i_), and mobilizes eCB from the postsynaptic CA1 PCs in a [Ca^2+^]_i_-dependent manner [32, 33]. Group 1 mGluR activates phospholipase C (PLC), which produces diacylglycerol (DAG), the precursor of 2-AG [34, 35], leading to the synthesis of 2-AG, thus mobilizing eCB in a PLCβ-dependent and [Ca^2+^]_i_-independent manner [36–38]. Importantly, concomitant activation of group 1 mGluR during postsynaptic depolarization has been shown to synergistically enhance PLCβ protein levels, resulting in an increase of eCB mobilization. This suggests a PLCβ-dependent synergistic enhancement of e-CB (S-eCB) [30, 33, 39].

Aβ pathogenesis has been shown to disrupt depolarization-induced suppression of inhibition (DSI) in CA1 PCs [40, 41], a phenomenon caused by [Ca^2+^]_i_-dependent eCB mobilization [32, 33]. As eCB mobilization is known to regulate the induction of hippocampal LTP [14, 15], Aβ-mediated disruption of [Ca^2+^]_i_-dependent eCB mobilization may have contributed to the impairment of hippocampal LTP and excitatory postsynaptic potential (EPSP)-spike (E-S) potentiation in a mouse model of AD [41]. However, the effect of AβO on PLCβ-dependent S-eCB mobilization and its relationship with LTP impairment in AD has not been reported.

Thus, understanding the effect of AβO on PLCβ-dependent S-eCB mobilization under in vivo-like conditions and how it consequently relates to LTP and behavioral memory impairments in mouse models of AD are critical for elucidating the molecular and synaptic mechanisms underlying memory impairments in AD. In this study, we addressed these questions by combining in vitro whole-cell patch-clamp recordings, western blot analysis, and an in vivo behavioral memory task. Using these methods, we characterized the impact of AβO on PLCβ-dependent S-eCB mobilization and its association with LTP and hippocampus-dependent memory impairments caused by amyloidosis in rat hippocampal slices in vitro and the 5XFAD mouse model of Alzheimer’s disease in vivo.

## Methods

### Animals

To test the acute effect of AβO on hippocampal S-eCB mobilization, hippocampal slices from Sprague-Dawley rats (SD, 2–3 weeks old, DaeHan Biolink, Korea) were used. Male and female rats were used in all in vitro experiments. To test the chronic effect of AβO, especially on hippocampus-dependent memory impairments caused by amyloidosis, the 5XFAD mouse model of AD (6–7 months old, #34840-JAX, Jackson Laboratory, USA), a mouse model of AD that mimics AβO depositions [42], was used. 5XFAD mice were used as SD rat models of AD are insufficient to mimic Aβ deposition and the amyloid hypothesis as yet [43–45]. C57BL/6 mice (6–7 months old, KOATECH, Korea) were used as wild type (WT) control mice for 5XFAD mice. Only male 5XFAD and C57BL/6 mice were used for all in vivo behavioral experiments. The 5XFAD mouse genotypes were identified by polymerase chain reaction (PCR) using genomic deoxyribonucleic acid (DNA) from the tails of the mice. All animal procedures followed the guidelines of the Institutional Animal Care and Use Committee of Korea University (guidelines for SD rats: KUIACUC-2017-103, guidelines for C57BL/6 and 5XFAD mice: KUIACUC-2019-0068).

### AβO preparation

Soluble AβO was prepared according to the methods described in a previous study [46]. Aβ_1-42_ (Aβ) and a scrambled form of Aβ as a control peptide [47] were purchased in powder form. Aβ and scrambled Aβ were dissolved for monomerization in 1,1,1,3,3,3-hexafluoro-2-propanol (HFIP, Sigma Aldrich, USA) at a final concentration of 1 mM, and the solution was incubated for 90 min. After the HFIP was evaporated, the remaining thin and clear film of Aβ or scrambled Aβ was dissolved in dimethyl sulfoxide (DMSO, Sigma Aldrich, USA) to prepare 5 mM Aβ or scrambled Aβ stocks, which were aliquoted and frozen at –20°C. Aβ or scrambled Aβ stocks were thawed and diluted to 100 μM in artificial cerebrospinal fluid (ACSF) and incubated for 18 h for oligomerization. The final AβO or scrambled AβO stock solutions was diluted to a final concentration of 200 nM in 31.2 ml ACSF, which was continuously bubbled and treated to rat hippocampal slices for 20 min in a recovery chamber before recording. In control experiments for normal brain conditions using the vehicle, rat hippocampal slices were treated with 0.004% DMSO in ACSF.

### Preparation of in vitro hippocampal slices

The brains of SD rats were removed following decapitation under deep isoflurane-induced anesthesia and transferred into ice-cold ACSF containing 126 mM NaCl, 3 KCl, 1.25 mM NaH_2_PO_4_, 2 mM MgSO_4_, 2 mM CaCl_2_, 25 mM NaHCO_3_, and 10 mM glucose at pH 7.2–7.4, which was continuously oxygenated with 95% O_2_/5% CO_2_. Horizontal hippocampal slices (350 μm thickness) were cut using a vibratome (VT1000S, Leica Biosystems, Germany) and immediately incubated for at least 1 h at room temperature in a submerged chamber perfused with oxygenated ACSF.

### In vitro eCB mobilization protocols

To quantify eCB mobilization, we measured the changes in eCB mobilization-induced Schaffer collateral (SC) stimulation-evoked inhibitory postsynaptic current (eIPSC) amplitudes from CA1 PCs through whole-cell voltage-clamp recordings, a method adopted in many studies investigating eCB mobilization [19, 29, 32, 48]. Whole-cell voltage-clamp recordings in CA1 PCs were performed using a MultiClamp 700B amplifier (Molecular Devices, USA) under the visual guidance of infrared differential interference contrast video microscopy (BW51W, Olympus, Japan), with the aid of a borosilicate electrode (tip resistance: 4–8 MΩ) that was filled with an intracellular solution containing 145 mM CsCl, 10 mM HEPES, 2 mM EGTA, 4 mM QX-314, 4 mM Mg-ATP, 0.4 mM NaCl-GTP, pH 7.2–7.3, and 270-280 mOsm/L. To record eIPSCs from the CA1 PCs, a stimulating electrode (A-M Systems, USA) was placed in the striatum radiatum to stimulate the SC pathway that carries CA3 PC axons. Electrical stimulation pulses of 200–400 μA amplitudes (20–40 μs) were generated using a constant current stimulator (model DS3, Digitimer Ltd., UK). eIPSCs were recorded in CA PCs every 1 s while maintaining the membrane potential at −70 mV. Before the application of CB1R antagonist AM251, DSI was induced by applying a voltage step from −70 mV to 0 mV for 1 s to detect the eCB-sensitive cells, which is a common method used in many studies [29, 32].

To induce S-eCB mobilization, the group 1 mGluR agonist (S)-3,5-dihydroxyphenylglycine (DHPG, 50 μM) was locally applied using a puff pipette located near the soma of CA1 PCs. To induce S-eCB mobilization, we combined group 1 mGluR activation with postsynaptic depolarization. In particular, instead of a physiologically unrealistic prolonged step depolarization (5–10 s) used in all studies inducing DSI [36, 41], a physiologically realistic postsynaptic depolarization was induced by mimicking in vivo-like sparse CA1 PC spikes (1 Hz) observed in vivo during memory processing [49, 50]. To this end, in vivo-like sparse CA1 PC spikes were mimicked by delivering 5 ms-voltage steps (from −70 to +40 mV) at 1 Hz for 60 s. DSI was measured by taking the mean amplitude of the first ten consecutive eIPSCs following the spikes and/or DHPG puff. In all voltage-clamp recordings, 6-cyano-7-nitroquinoxaline-2,3-dione (CNQX, 20 μM) and D-2-amino-5-phosphonovaleric acid (D-AP5, 50 μM) were applied to prevent ionotropic glutamatergic currents and synaptic plasticity. To test group 1 mGluR subtype-dependence of PLCβ-dependent S-eCB mobilization, the experiments were repeated in the presence of an mGluR1 antagonist, LY367385 (100 μM), or an mGluR5 antagonist, 2-methyl-6-(phenylethynyl) pyridine (MPEP, 10 μM). All data were filtered at 2 kHz and digitized at 5 kHz using an ITC-18 AD board (HEKA Elektronik, Germany). Igor Pro software (WaveMetrics, USA) was used to generate command signals and analyze the data.

### S-eCB mobilization induced spike timing-dependent potentiation (tLTP) protocol

To directly test whether S-eCB mobilization can induce hippocampal tLTP, the PLCβ-dependent S-eCB mobilization experimental protocol was modified to introduce presynaptic CA3 PC spikes during the S-eCB mobilization protocol to induce tLTP. Whole-cell current-clamp recordings were made from hippocampal CA1 PCs using a glass electrode (tip resistance: 5–8 MΩ) filled with intracellular solution containing 110 mM potassium gluconate, 4 mM NaCl, 40 mM HEPES, 4 mM Mg-ATP, 0.3 mM NaCl-GTP, and 270–280 mOsm/L, at pH of 7.2–7.3. Two stimulating electrodes were positioned on the stratum radiatum of the hippocampal CA1 area: one was used for monitoring EPSPs in the test pathway and the other for monitoring EPSPs in the control pathway. The tLTP induction protocol was implemented only in the test pathway. To directly test how S-eCB mobilization is related to tLTP induction, the tLTP induction paradigm was designed by modifying our S-eCB mobilization protocol. Here, SC stimulation-evoked presynaptic CA3 PC spikes were introduced during the S-eCB mobilization protocol: 1 Hz presynaptic CA3 PC spikes were evoked 10 ms before the 1 Hz postsynaptic CA1 PC spikes during the activation of mGluR5, to induce tLTP. This ensured that PLCβ-dependent S-eCB mobilization was induced during the pairing of presynaptic CA3 PC spikes and postsynaptic CA1 PC spikes for tLTP induction at the CA3-CA1 excitatory synapses. During the baseline EPSP recordings, SC stimulation-evoked EPSPs were evoked every 6 s for at least 10 min. After a stable baseline was established, tLTP was induced in which SC stimulation-evoked presynaptic CA3 PC spikes were paired with postsynaptic CA1 PC spikes evoked by current pulses (800 pA, 3 ms current steps) with a 10 ms time window at 1 Hz. This was repeated 200 times during the activation of mGluR5 through the application of DHPG (50 μM) [51] in the presence of LY367385 (100 μM). SC stimulation-evoked EPSP responses were recorded for at least 30 min after the tLTP induction protocol. In all tLTP induction experiments, the membrane potentials of the CA1 PCs were held at −70 mV. To analyze the changes in synaptic efficacy following tLTP induction, the EPSP slope was measured using a linear fit on the rising slope of the EPSP between the time points that were 20–25% and 75–80% from the EPSP peak amplitude during the baseline conditions. Changes in synaptic efficacy by the tLTP induction protocol were estimated as percentage changes relative to the mean EPSP slope of the 10-min baseline. To compare the synaptic efficacy changes across different neurons and experimental conditions, the mean of the normalized EPSP slopes in the time period of 25–30 min after the end of the tLTP induction protocol was used [51].

### Western blotting

Western blotting was performed according to the methods described by Mahmood and Yang [52], with slight modifications according to the manufacturer’s recommendations. Hippocampal PLCβ1 of SD rat, C57BL/6, or 5XFAD mice were prepared from homogenized hippocampi dissolved in 200 μL of RIPA buffer (Bio-Rad, USA), which was resolved on non-reducing 10% tris-glycine-sodium dodecyl sulfate-polyacrylamide gel electrophoresis (SDS-PAGE) gels (Bio-Rad, USA) with 4× Laemmli sample buffers (Bio-Rad, USA) [53]. The gels were transferred onto 0.2-μm PVDF membranes (Bio-Rad, USA) according to the manufacturer’s recommendations. The membranes were blocked in 5% bovine serum albumin (BSA) in Tris-buffered saline containing 0.01% Tween 20 (TBST, Bio-Rad, USA) for 1 h at room temperature. Blots were incubated with the primary antibody of PLCβ1 (0.4 μg/mL, NBP2-38220, Novus, USA) in wash buffer containing 5% BSA overnight at 4°C. After incubation with the primary antibody, the membranes were rinsed three times with TBST buffer and then incubated with secondary horseradish peroxidase (HRP)-conjugated anti-rabbit antibodies (1:3000, Cat# 170-6515, Control# 64170140, RRID: AB_2617112, Bio-Rad, USA) in wash buffer containing 5% BSA for 1 h at room temperature. Immunoreactivity was detected with enhanced chemiluminescence (Bio-Rad, USA) using the Fluorchem E system (ProteinSimple, USA) and analyzed with ImageJ software. Molecular weight values were estimated using the Precision Plus Protein™ Dual Color Standards (Bio-Rad, USA).

### Stereotaxic drug injection

For stereotaxic drug injections, C57BL/6 or 5XFAD mice were deeply anesthetized with 2% isoflurane (2 mL/min flow rate) and head-fixed into a stereotaxic frame (Stoelting Co., USA). Craniotomies were made at four sites to target the CA1 areas of the ventral and dorsal hippocampi (ventral hippocampus; from bregma: anteroposterior −3 mm, lateral ± 2.70 mm, and dorsoventral −4.2 mm. Dorsal hippocampus; from bregma: anteroposterior −1.7 mm, lateral ± 1.2 mm, and dorsoventral −1.4 mm) [54]. This was in order to inject 0.5 μl DMSO (0.05%) as the vehicle or the PLC activator, m-3M3FBS (50 μM), at a rate of 0.1 μl/min through a Hamilton syringe using a motorized stereotaxic injector (Stoetling Co., USA). The syringe was left in the brain for more than 5 min to allow for drug diffusion, and the scalp was sutured and disinfected with antibiotics. Mice were returned to their home cage for recovery for three days, after which they underwent contextual fear conditioning.

### Contextual fear conditioning (CFC) protocol

CFC was performed in conditioning chambers (Coulbourn Instruments, USA) consisting of metal panel sidewalls, Plexiglas front and rear walls, and a stainless-steel grid floor composed of 16 grid bars. The grid floor was connected to a precision animal shocker (Coulbourn Instruments, USA) set to deliver a 0.5 mA foot shock for 1 s. A ceiling-mounted video camera recorded the behavior activity, which fed the video into a customized computer software (MATLAB, USA). The chambers were cleaned with 70% ethanol solution prior to animal placement. For habituation on day 1, C57BL/6 or 5XFAD mice were placed in the conditioning chamber for 20 min of free exploration (habituation). On day 2, the day after the habituation session, C57BL/6 or 5XFAD mice were placed into the conditioning chamber for the fear conditioning session (483 s), consisting of a 120-s baseline period followed by three 0.5 mA, 1-s-long foot shocks (interstimulus interval equal to 120 s; conditioning). On day 3, the day after the conditioning session, the mice were placed back into the conditioning chamber for 2 min to assess their memory in the electric shock-free condition. Behavioral mobility data collected during the contextual conditioning experiments were automatically detected using the gray scaling method of EthoVision XT (Noldus, USA), which uses light and dark thresholds to determine the subject within the conditioning chamber. Because freezing is considered a conditioned response to CFC during the recall session, freezing was detected when the threshold was below the 2.0% threshold of mobility, which means there could be no more than a 2.0% change in the pixels of a detected mouse between the current sample and the previous sample [55]. Percentage freezing was estimated by scoring freezing behavior, defined as the absence of movement except that it required respiration for at least 2 s.

### Drugs

The group 1 mGluR agonist DHPG (50 μM, Tocris, UK) was used to activate group 1 mGluR. To block mGluR5 and mGluR1a, MPEP (10 μM, Tocris, UK) and LY367385 (100 μM, Tocris, UK) was applied to rat hippocampal slices, respectively. AM251 (3 μM, Tocris, UK) was used to block presynaptic CB1R. U73122 (5 μM, Tocris, UK) was used to block PLCβ activity. m-3M3FBS (30 μM, Tocris, UK) was used as the PLC activator. The NMDAR antagonist D-AP5 (50 μM, Tocris, UK) and the AMPA receptor antagonist CNQX (20 μM, Tocris, UK) were used for the eIPSC recordings. AβO and scrambled AβO were synthesized from a lyophilized powder of Aβ and scrambled Aβ peptide, respectively (Bachem, Japan). A 4× Laemmli sample buffer (Bio-Rad, USA) and running buffer (Bio-Rad, USA) were used for western blot SDS-PAGE. For the antibody incubation step in western blotting, rabbit monoclonal primary antibodies (NBP2-38220, Novus, USA) and HRP-conjugated secondary anti-rabbit antibodies (Cat# 170-6515, Control# 64170140, RRID: AB_2617112, Bio-Rad, USA) were used, respectively.

### Statistical analysis

All data are expressed as mean ± standard error of the mean (SEM). Statistical significance was measured using Student’s *t* test or one-way analysis of variance (ANOVA) followed by a post hoc Tukey’s test. Statistical significance was set at *p* < 0.05.

## Results

### Aβ oligomers inhibit synergistic enhancement of eCB (S-eCB) mobilization in hippocampal CA1 pyramidal cells

**Fig. 1:**
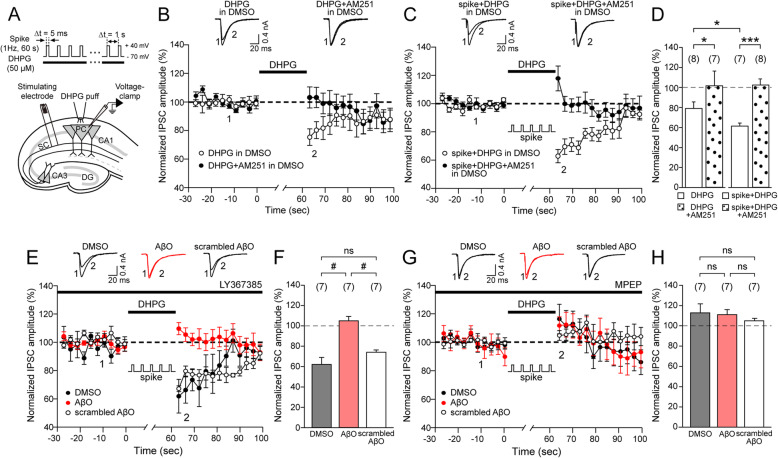
AβO disrupts synergistic enhancement of endocannabinoid (S-eCB) mobilization. **a** Schematic of the S-eCB mobilization protocol: whole-cell voltage-clamp recording in CA1 pyramidal cells (PCs) to measure Schaffer collateral (SC) stimulation-evoked inhibitory postsynaptic potentials (eIPSCs) in rat hippocampal slices in vitro. S-eCB mobilization was induced by postsynaptic CA1 PC spikes (spike, at 1 Hz) with DHPG (50 μM) for 60 s. **b**, **c** Time course of the changes in mean eIPSC amplitudes following DHPG (empty circle, *n* = 8, **b**), DHPG + AM251 (3 μM) (filled circle, *n* = 7, **b**), spike + DHPG (empty circle, *n* = 7, **c**), and spike + DHPG + AM251 (filled circle, *n* = 8, **c**) in DMSO-treated rat hippocampal slices. **d** Mean of normalized eIPSC amplitudes of the first 10 s after S-eCB mobilization protocol in **b** and **c**. DHPG or spike + DHPG (empty bar), DHPG + AM251, or spike + DHPG + AM251 (dotted bar). **e** Same as **c**, but in the presence of LY367385 (100 μM) in DMSO-treated (black circle, *n* = 7), AβO-treated (red circle, *n* = 7), and scrambled AβO-treated rat hippocampal slices (empty circle, *n* = 7). **f** The mean of the normalized eIPSC amplitudes in **e**. DMSO (black bar), AβO (red bar), and scrambled AβO (empty bar). **g–h** Same as **e** and **f**, but in the presence of MPEP (10 μM) in DMSO-treated (black, *n* = 7), AβO-treated (red, *n* = 7), and scrambled AβO-treated rat hippocampal slices (empty, *n* = 7). Inset: **b**, **c**, **e**, and **g** representative eIPSC traces at the indicated time points (1, 2) in each condition. Statistical tests: **d** Unpaired Student’s *t* test, *p < 0.05, ***p < 0.001; **f**, **h** one-way ANOVA with post hoc Tukey’s test, #*p* < 0.05, ns: *p* > 0.05. Data are represented as mean ± SEM

To investigate the effect of AβO on PLCβ-dependent eCB and S-eCB mobilization in CA1 PCs, we established a protocol that can measure S-eCB mobilization induced by a concomitant activation of group 1 mGluR during postsynaptic depolarization. We performed whole-cell voltage-clamp recordings in CA1 PCs from rat hippocampal slices and recorded eIPSC amplitudes (Fig. 1a) before and after activation of group 1 mGluR using the group 1 mGluR agonist DHPG (50 μM) [31, 32], or with concomitant activation of group 1 mGluR with postsynaptic depolarization [30, 36], respectively (Fig. 1b–d). The DHPG puff alone induced the suppression of the eIPSC amplitudes (empty circle, Fig. 1b), where the first ten eIPSC amplitudes decreased to 79 ± 7% of the mean eIPSC amplitudes measured at the baseline before the DHPG puff (empty bar, Fig. 1d left). This was blocked by the CB1R antagonist AM251 (3 μM) (filled circle, Fig. 1b; 101 ± 7%, dotted bar, *p* < 0.05, unpaired Student’s *t* test, Fig. 1d left, indicating that the activation of group 1 mGluR could induce eCB mobilization, similar to the findings of previous reports [36, 56]. Next, we investigated whether pairing DHPG puff with 60-s-long in vivo-like postsynaptic spike trains at 1 Hz could induce S-eCB mobilization in DMSO-treated rat hippocampal slices. eIPSC amplitudes were suppressed (empty circle, Fig. 1c) to 62 ± 4% of the baseline eIPSC amplitudes (empty bar, Fig. 1d right), which was also completely blocked by AM251 (filled circle, Fig. 1c; 103 ± 6%, dotted bar, *p* < 0.001, unpaired Student’s *t* test, Fig. 1d right). In fact, DHPG puff during in vivo-like sparse spike trains induced synergistic enhancement in the suppression of eIPSC amplitudes (empty bar, Fig. 1d right) compared to those seen in the DHPG puff-only condition (empty bar, *p* < 0.05, unpaired Student’s *t* test, Fig. 1d left), indicating that our in vivo-like spike trains during group 1 mGluR activation can induce S-eCB mobilization.

Group 1 mGluRs consist of two subtypes in the hippocampus, mGluR1 and mGluR5 [57, 58]; therefore, to determine which subtype of group 1 mGluRs mainly induces S-eCB mobilization, we repeated the S-eCB mobilization experiment in the presence of either an mGluR1 antagonist, LY367385 (100 μM), or an mGluR5 antagonist, MPEP (10 μM) in DMSO-treated, AβO-treated, and scrambled AβO-treated rat hippocampal slices (Fig. 1e–h). In the presence of LY367385, the eIPSC amplitudes following 60-s-long DHPG puff during 1 Hz spikes decreased in both DMSO-treated rat hippocampal slices (filled circle, Fig. 1e; 62 ± 7%, black bar, Fig. 1f) and in scrambled AβO-treated rat hippocampal slices (empty circle, Fig. 1e; 75 ± 2%, empty bar, Fig. 1f). However, the suppression of eIPSC amplitudes was completely blocked in AβO-treated rat hippocampal slices (red circle, Fig. 1e; 106 ± 4%, red bar, Fig. 1f), which was statistically different from those recorded in DMSO-treated rat hippocampal slices and scrambled AβO-treated rat hippocampal slices (DMSO vs. AβO, *p* < 0.05, AβO vs. scrambled AβO, *p* < 0.05, one-way ANOVA with post hoc Tukey's test, Fig. 1f). When the experiment was repeated in the presence of MPEP, the eIPSC amplitudes following DHPG puff during 1 Hz spikes did not change in the DMSO-treated (filled circle, Fig. 1g; 113 ± 10%, black bar, Fig. 1h), AβO-treated (red circle, Fig. 1g; 112 ± 5%, red bar, Fig. 1h), or scrambled AβO-treated rat hippocampal slices (empty circle, Fig. 1g; 106 ± 2%, empty bar, Fig. 1h), and they were not statistically different (*p*﻿ > 0.05, one-way ANOVA with post hoc Tukey’s test, Fig. 1h). Overall, our results indicate that in vivo-like spike trains during mGluR5 activation, but not mGluR1, can induce S-eCB mobilization, as demonstrated by the synergistic enhancement in the suppression of eIPSC amplitudes. We also showed, for the first time, that such S-eCB mobilization is impaired specifically by AβO as scrambled AβO spared S-eCB mobilization.

### AβO reduces PLCβ to impair S-eCB mobilization in hippocampal CA1 pyramidal cells

**Fig. 2:**
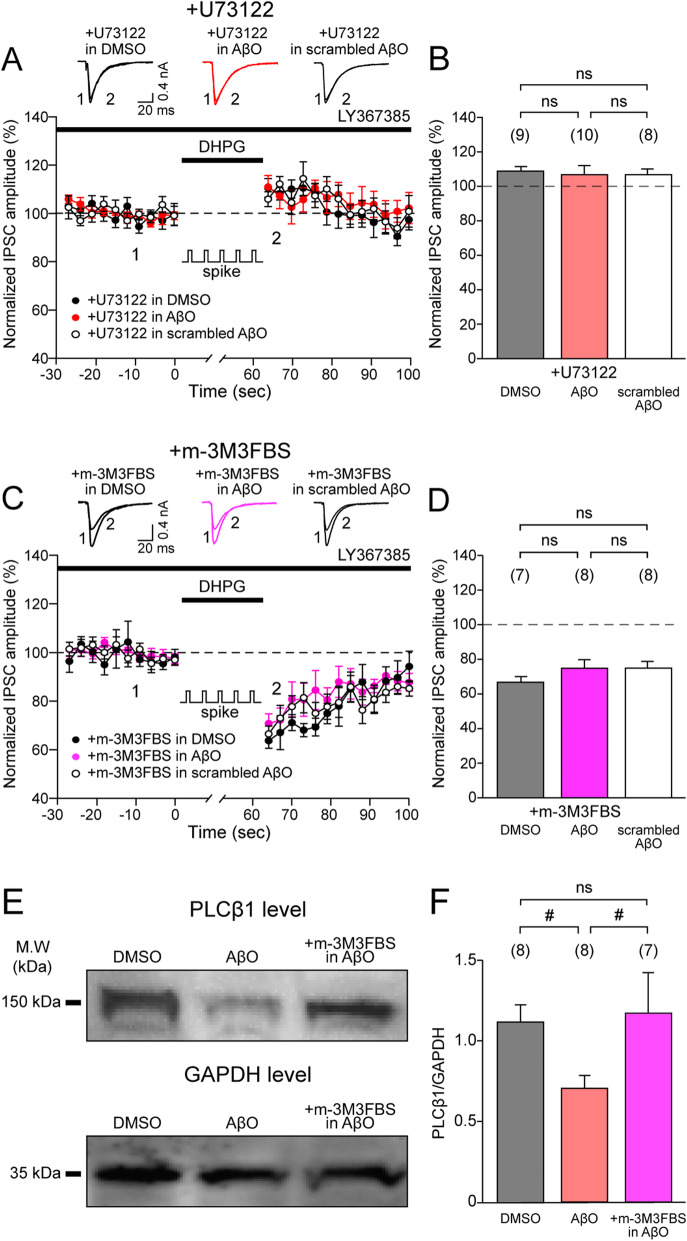
m-3M3FBS, a PLC activator, restores PLCβ-dependent S-eCB mobilization and PLCβ1 protein levels disrupted by AβO. **a** Time courses of the changes in mean eIPSC amplitudes following a S-eCB mobilization paradigm consisting of 60-s-long postsynaptic CA1 PC spikes (spike, at 1 Hz) with DHPG (50 μM) and LY367385 (100 μM) application in the presence of a PLCβ blocker, U73122 (5 μM) (+ U73122) in DMSO-treated (black circle, *n* = 9), AβO-treated (red circle, *n* = 10), and scrambled AβO-treated rat hippocampal slices (empty circle, *n* = 8). **b** The mean of the normalized eIPSC amplitude of the first 10 s after S-eCB mobilization protocol in rat hippocampal slice treated with DMSO (black bar), AβO (red bar), and scrambled AβO (empty bar). **c**–**d** Same as **a** and **b** but for eIPSCs in the presence of a PLC activator, m-3M3FBS (30 μM) (+ m-3M3FBS) in DMSO-treated (black, *n* = 7), AβO-treated (magenta, *n* = 8), and scrambled AβO-treated rat hippocampal slices (empty, *n* = 8). Inset: **a**, **c** representative eIPSC traces at indicated time points (1, 2). **e** Representative photomicrograph of western blots of PLCβ1 (top, 150 kDa) and GAPDH (down, 35 kDa) proteins from DMSO-treated (left lane), AβO-treated (middle lane) rat hippocampal slices, and in the presence of m-3M3FBS (30 μM) in AβO-treated rat hippocampal slices (right lane). **f** PLCβ1 protein levels normalized to the GAPDH protein levels in DMSO-treated (black, *n* = 8), AβO-treated rat hippocampal slices (red, *n* = 8), and in the presence of m-3M3FBS AβO-treated rat hippocampal slices (magenta, *n* = 7). Statistical tests: **b**, **d**, **f** One-way ANOVA with post hoc Tukey’s test, # *p* < 0.05, ns: *p* > 0.05. Data are represented as mean ± SEM

PLCβ isozymes that are involved in eCB mobilization have been suggested to act as sensors for detecting the co-activation of postsynaptic depolarization and mGluR5 activation [17, 33, 39]. Given that S-eCB mobilization is disrupted by AβO, it may be that PLCβ or the downstream chemical cascade that is involved in eCB production is affected by AβO. To directly investigate this hypothesis, we applied the PLCβ blocker, U73122 (5 μM), and repeated the S-eCB mobilization experiment, as shown in Fig. 1e, in DMSO-treated, AβO-treated, and scrambled AβO-treated rat hippocampal slices. We found that U73122 completely blocked the synergistic enhancement in the suppression of eIPSC amplitudes in DMSO-treated rat hippocampal slices (black circle, Fig. 2a; 110 ± 3%, black bar, Fig. 2b), which was similar to those observed in the AβO-treated (red circle, Fig. 2b; 107 ± 5%, red bar, *p* > 0.05, one-way ANOVA with post hoc Tukey’s test, Fig. 2b) and in the scrambled AβO-treated rat hippocampal slices (empty circle, Fig. 2a; 108 ± 3%, empty bar, *p* > 0.05, one-way ANOVA with post hoc Tukey’s test, Fig. 2b). These results indicate that S-eCB mobilization that is disrupted by AβO is completely occluded by U73122, confirming our hypothesis that AβO could have blocked S-eCB mobilization in CA1 PCs by interfering with the PLCβ pathway. Because a decrease in PLCβ interferes with S-eCB mobilization in AβO-treated rat hippocampal slices (Fig. 2a, b), we hypothesized that an increase in PLCβ activity using a PLC activator, m-3M3FBS, would be able to restore AβO-induced impairment of S-eCB mobilization. To test this hypothesis, the S-eCB mobilization experiment was repeated in the presence of m-3M3FBS (30 μM) in DMSO-treated, AβO-treated, and scrambled AβO-treated rat hippocampal slices. Surprisingly, in the presence of m-3M3FBS, synergistic enhancement of the suppression of eIPSC amplitudes could be fully restored in the AβO-treated rat hippocampal slices (magenta circle, Fig. 2c; 75 ± 5%, magenta bar, Fig. 2d), similar to the levels recorded from the DMSO-treated (black circle, Fig. 2c; 68 ± 4%, black bar, Fig. 2d) and the scrambled AβO-treated rat hippocampal slices (empty circle, Fig. 2c; 73 ± 4%, empty bar, Fig. 2d), which were not significantly different (*p* > 0.05, one-way ANOVA with post hoc Tukey’s test, Fig. 2d). Together, these findings indicate that PLCβ is impaired by AβO to disrupt S-eCB mobilization, and increasing PLC activation using m-3M3FBS can restore S-eCB mobilization disrupted by AβO. To ascertain that the reduction in hippocampal PLCβ protein expression is mediated by AβO, especially that of PLCβ1, which is enriched in the hippocampus [59] and is known to be involved in mGluR-induced eCB mobilization [17, 33], a western blot analysis of PLCβ1 was carried out (Fig. 2e). The protein expression levels of PLCβ1 in the AβO-treated rat hippocampal slices significantly decreased compared to those in the DMSO-treated rat hippocampal slices (PLCβ1/glyceraldehyde-3-phosphate dehydrogenase (GAPDH); DMSO: 1.13 ± 0.11, AβO: 0.72 ± 0.08, *p* < 0.05, one-way ANOVA with post hoc Tukey’s test, Fig. 2e, f). However, treatment with m-3M3FBS fully restored the PLCβ1 protein levels in the AβO-treated rat hippocampal slices, similar to the results seen in the DMSO-treated rat hippocampal slices (PLCβ1/GAPDH; AβO + m-3M3FBS: 1.18 ± 0.26, *p* > 0.05, one-way ANOVA with post hoc Tukey’s test, Fig. 2e, f). Together, these results show that AβO disrupts PLCβ-dependent eCB mobilization by directly reducing the protein expression levels of PLCβ1.

### Activation of PLCβ restores AβO-induced impairment of spike-timing-dependent potentiation at hippocampal CA3-CA1 synapses

**Fig. 3:**
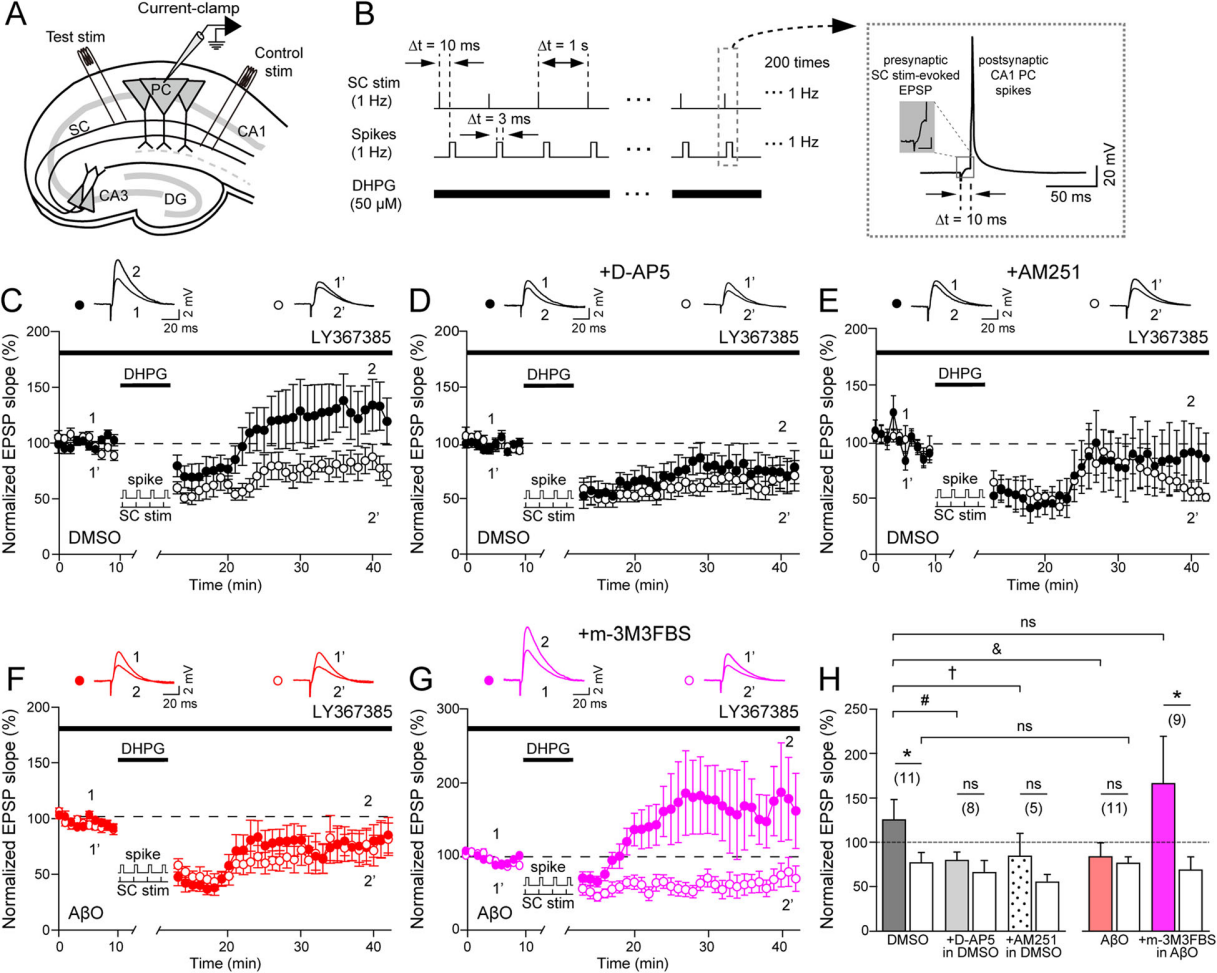
m-3M3FBS restores spike-timing-dependent potentiation (tLTP) impaired by AβO. **a** Experimental schematic. Whole-cell current-clamp recordings in CA1 pyramidal cells (PCs) and Schaffer collateral (SC) stimulation for tLTP induction at CA3-CA1 excitatory synapses in rat hippocampal slices in vitro. **b** tLTP induction paradigm consisting of S-eCB mobilization protocol with SC stimulation-evoked presynaptic CA3 PC spikes (SC stim, top) 10 ms before postsynaptic CA1 PC spikes (spikes, middle), repeated 200 times at 1 Hz with DHPG (50 μM) application (bottom). Boxed inset: enlarged presynaptic SC stim-evoked EPSPs paired with postsynaptic CA1 PC spikes during tLTP induction. **c**–**g** EPSP slopes normalized to the mean of the 10-min baseline after tLTP in DMSO-treated slices (*n* = 11, **c**), in the presence of D-AP5 (50 μM) in DMSO-treated slices (*n* = 8, **d**), in the presence of AM251 (3 μM) in DMSO-treated slices (*n* = 5, **e**), in AβO-treated slices (*n* = 11, **f**), and in the presence of m-3M3FBS (30 μM) in AβO-treated slices (*n* = 9, **g**). Filled circles: test pathways, empty circles: control pathways. Inset: representative EPSP traces at indicated time points (1, 2 or 1’, 2’). **h** The mean of normalized EPSPs slopes from the last 5 min of the test (filled bars) and control pathways (empty bars) in DMSO (black), + D-AP5 in DMSO-treated slices (gray), + AM251 in DMSO-treated slices (dotted), in AβO-treated slices (red), and + m-3M3FBS in AβO-treated slices (magenta). Statistical tests: **h** Paired Student’s *t* test for comparison between the test and control pathways within the same group, *p < 0.05, ns: *p* > 0.05; one-way ANOVA with post hoc Tukey’s test for comparison among the test pathways in five different groups (DMSO vs. + D-AP5 in DMSO, # *p* < 0.05; DMSO vs. + AM251 in DMSO, † *p* < 0.05; DMSO vs. AβO, & *p* < 0.05; DMSO vs. AβO + m-3M3FBS, ns: *p* > 0.05). Data are represented as mean ± SEM

To directly test whether there are causal links between AβO-induced disruption of S-eCB mobilization and impairment of LTP induction by AβO, presynaptic CA3 PC spikes were introduced during the S-eCB mobilization protocol. This ensured S-eCB mobilization while presynaptic CA1 PC and CA3 PC spikes were paired at a 10 ms time window to induce tLTP (Fig. 3a, b). This S-eCB mobilization-ensuring tLTP protocol induced robust potentiation of EPSP slopes in the test pathway compared to the control pathway (test: 127 ± 22%, control: 81 ± 10%; paired Student’s *t* test, Fig. 3c, h). In fact, mGluR5-mediated long-term depression (LTD) was induced in the control pathway, which is consistent with previous studies demonstrating that activation of mGluR5 induces LTD at the CA3-CA1 synapse [60, 61]. tLTP was completely blocked by D-AP5 (50 μM) (Fig. 3d) and AM251 (3 μM) (Fig. 3e) while mGuR5-mediated LTD was induced in both the test and control pathways (D-AP5, test: 66 ± 13%, control: 67 ± 11%, *p* > 0.05, paired Student’s *t* test, Fig. 3d, h; AM251, test: 87 ± 25%, control: 58 ± 8%, *p* > 0.05, paired Student’s *t* test, Fig. 3e, h). These results indicate that S-eCB mobilization is indeed required for the induction of NMDAR-dependent tLTP, while mGluR5-mediated LTD is NMDAR and CB1R-independent. When we repeated the tLTP induction in AβO-treated rat hippocampal slices, we found that AβO completely blocked the induction of tLTP in the test pathway (test: 86 ± 13%, control: 78 ± 13%, *p* > 0.05, paired Student’s *t* test, Fig. 3f, h), whereas mGluR-LTD in the control pathway was unaffected by AβO when compared to the control pathways in AβO-treated and DMSO-treated rat hippocampal slices (control in DMSO: 81 ± 10%, control in AβO: 78 ± 13%, *p* > 0.05, unpaired Student’s *t* test, Fig. 3c, f, h). These results show that while sparing mGluR5-mediated LTD, AβO specifically impaired NMDAR-dependent tLTP by disrupting S-eCB mobilization. Because increasing PLC activity restored S-eCB mobilization (Fig. 2c, d), we hypothesized that m-3M3FBS could also restore tLTP that was impaired by AβO. Surprisingly, bath application of m-3M3FBS (30 μM) in AβO-treated rat hippocampal slices fully restored tLTP (test: 169 ± 50%, control: 70 ± 13%, *p* < 0.05, paired Student’s *t* test, Fig. 3g, h), similar to the level observed in the DMSO-treated rat hippocampal slices (*p* > 0.05, one-way ANOVA with post hoc Tukey’s test, Fig. 3c, g, h). These results show, for the first time, that enhancing PLCβ-dependent S-eCB mobilization by directly increasing the PLCβ1 protein levels can restore hippocampal tLTP impaired by acute application of AβO.

### Activation of PLCβ restores hippocampal PLCβ1 protein levels and contextual fear memory in 5XFAD mice

**Fig. 4:**
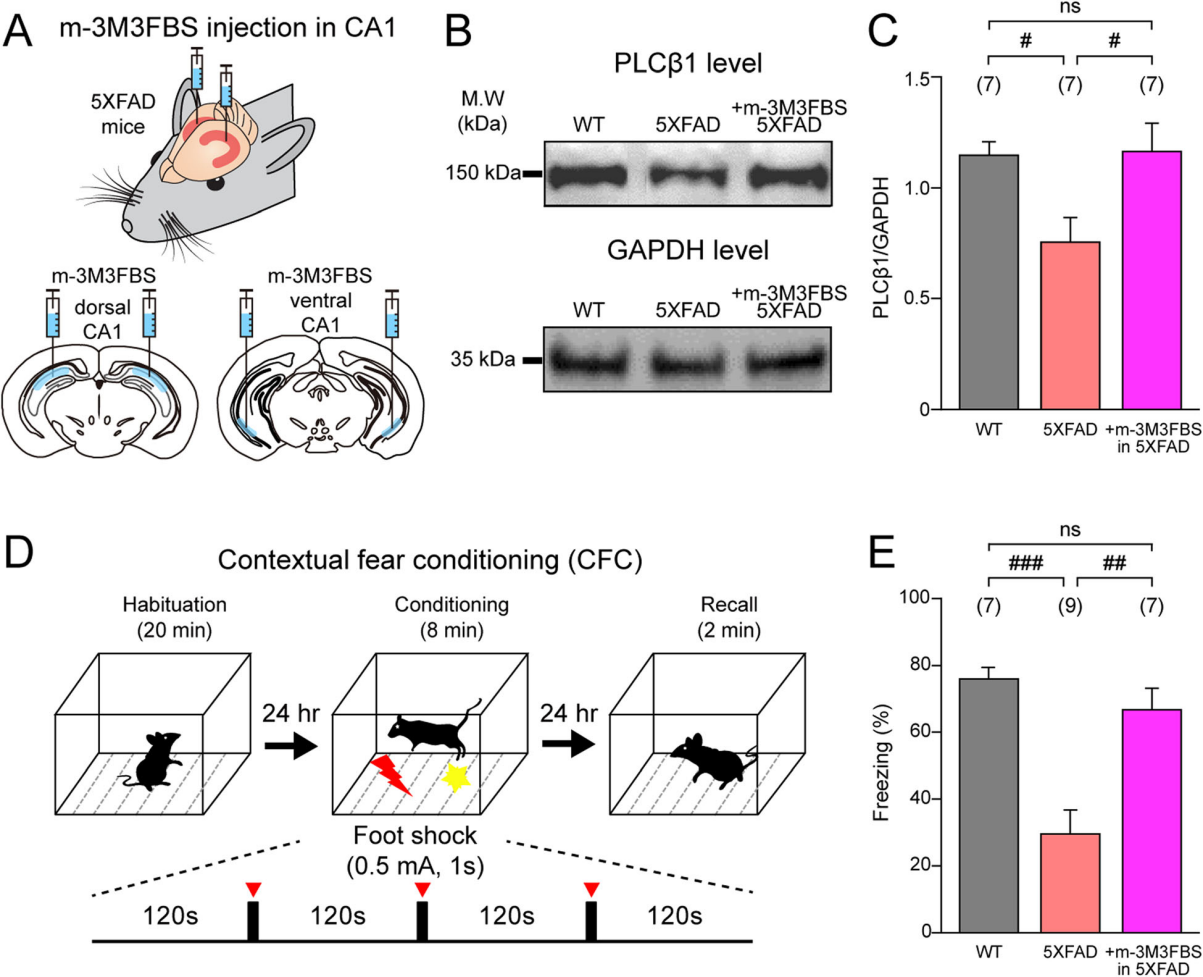
m-3M3FBS restores hippocampus-dependent contextual fear memory impaired in 5XFAD mice. **a** Experimental schematic. DMSO or m-3M3FBS (50 μM) was injected into the dorsal and ventral hippocampal CA1 regions of wild type (WT) control mice and 5XFAD mice. **b** Representative photomicrograph of western blots of PLCβ1 protein (top, 150 kDa) and GAPDH protein (bottom, 35 kDa) in WT mice (left lane), 5XFAD mice (middle lane), and m-3M3FBS-injected 5XFAD mice (+ m-3M3FBS in 5XFAD, right lane). **c** PLCβ1 protein levels normalized to GAPDH protein levels in WT mice (black, *n* = 7), 5XFAD mice (red, *n* = 7), and m-3M3FBS-injected 5XFAD mice (magenta, *n* = 7). **d** Experimental schematic for contextual fear conditioning (CFC). CFC was performed 3 days after DMSO or m-3M3FBS injection. On day 1, mice were habituated for 20 min in the conditioning chamber (habituation). On day 2, electrical foot shocks (0.5 mA, 1s) were delivered three times every 120 s (conditioning). On day 3, the mice were returned to the conditioning chamber for memory recall test for 2 min (Recall). **e** The mean of the percentage of freezing, a fear memory index, in WT mice (black, *n* = 7), 5XFAD mice (red, *n* = 9), and m-3M3FBS-injected-5XFAD mice (magenta, *n* = 7). Statistical tests: **c**, **e** One-way ANOVA with post hoc Tukey’s test, # *p* < 0.05, ## *p* < 0.01, ### *p* < 0.001, ns: > 0.05. Data are represented as mean ± SEM

Considering that increasing the PLCβ1 protein levels can restore AβO-impaired tLTP, and that tLTP is the most probable synaptic mechanism underlying learning and memory [62–64], we hypothesized that increasing PLCβ1 protein levels could also restore behavioral memory impairment in AD. We used the 5XFAD mouse model of AD, which is known as a representative rodent model that mimics AβO pathophysiology in AD [42], to examine whether (1) PLCβ1 protein levels are affected in 5XFAD mice and if so, whether these can be restored pharmacologically using m-3M3FBS and (2) whether contextual fear memory, a well-known hippocampal-dependent behavioral assay [65, 66] which is known to be impaired in 5XFAD mice [67, 68], can also be restored by PLCβ activation. To this end, we injected either DMSO in WT mice (C57BL/6) and 5XFAD mice for control experiments or m-3M3FBS in 5XFAD mice in both the dorsal and ventral regions of the bilateral hippocampi (Fig. 4a). Three days after injection, we performed western blot analyses of PLCβ1 and noted that the PLCβ1 protein levels in mouse hippocampal slices from DMSO-injected 5XFAD mice were significantly decreased compared to those in DMSO-injected WT mice (PLCβ1/GAPDH; WT: 1.16 ± 0.06, 5XFAD: 0.76 ± 0.11, *p* < 0.05, one-way ANOVA with post hoc Tukey’s test, Fig. 4b, c), consistent with the acute treatment of AβO in rat hippocampal slices (Fig. 2e, f). However, in hippocampal slices cut from 5XFAD mice injected with m-3M3FBS, the levels of PLCβ1 protein were fully restored compared to those observed in the hippocampal slices cut from DMSO-injected WT mice (5XFAD + m-3M3FBS: 1.17 ± 0.13, *p* > 0.05, one-way ANOVA with post hoc Tukey’s test, Fig. 4b, c). These results suggest that hippocampal PLCβ1 protein levels are significantly decreased by acute treatment with AβO, not only in rat hippocampal slices (Fig. 2e, f) but also in the hippocampi of chronic stages of AD in 5XFAD mice. In addition, it was found that m-3M3FBS fully restored PLCβ1 protein levels back to normal. Next, to determine whether a decrease in PLCβ1 protein levels correlates with hippocampus-dependent memory impairment in a mouse model of AD, we performed a contextual fear memory task in the DMSO-injected WT, DMSO-injected 5XFAD, and m-3M3FBS-injected 5XFAD mice (Fig. 4d). The freezing response in 5XFAD mice during memory recall was significantly lower than that in DMSO-injected WT mice (DMSO-injected WT: 76 ± 3%, 5XFAD: 36 ± 6%, *p* < 0.001, one-way ANOVA with post hoc Tukey’s test, Fig. 4e), which is consistent with many studies showing impairment of contextual fear memory in 5XFAD mice [67, 68]. We then determined whether a PLC activator could rescue contextual fear memory impairment in m-3M3FBS-injected 5XFAD mice. Interestingly, m-3M3FBS-injected 5XFAD mice showed significantly increased freezing responses (67 ± 6%, Fig. 4e) compared to DMSO-injected 5XFAD mice (*p* < 0.01, one-way ANOVA with post hoc Tukey’s test, Fig. 4e), which was similar to the level observed in the DMSO-injected WT mice (*p* > 0.05, one-way ANOVA with post hoc Tukey’s test, Fig. 4e). Together, these results indicate that PLCβ1 protein levels are related to successful memory recall and that increasing PLCβ1 protein levels could restore contextual fear memory impairment in AD mice.

## Discussion

In this study, we show that AβO disrupts PLCβ-dependent S-eCB mobilization by decreasing hippocampal PLCβ1 protein levels, which in turn impairs hippocampal NMDAR-mediated tLTP at CA3-CA1 excitatory synapses in rat hippocampal slices acutely treated with AβO in vitro. Pharmacological activation of PLCβ1 using the PLC activator, m-3M3FBS, reinstated PLCβ1 protein levels, which consequently fully restored S-eCB mobilization and tLTP in rat hippocampal slices in vitro. Moreover, we found that hippocampal PLCβ1 protein levels in the 5XFAD mouse model of AD were also decreased and that direct hippocampal injection of m-3M3FBS in vivo in the 5XFAD mice fully restored the hippocampus-dependent contextual fear memory impairment in 5XFAD mice. Based on our results, we demonstrate, for the first time, the role of PLCβ-dependent S-eCB mobilization in synaptic plasticity and behavioral memory in healthy control rodents and a rodent model of AD.

To date, AβO has been shown to disrupt only [Ca^2+^]_i_-dependent eCB mobilization induced by physiologically unrealistic prolonged and strong depolarization by monitoring the DSI of eIPSCs in CA1 PCs [32, 41]. However, the effect of AβO on PLCβ-dependent S-eCB mobilization induced by mGluR activation during physiologically realistic postsynaptic depolarization has not been investigated. This is an important issue, considering that PLCβ has already been suggested to function as a sensor for detecting the co-activation of postsynaptic depolarization and mGluR5 [17, 33, 39]. In this study, we first established a physiologically realistic paradigm for inducing PLCβ-dependent S-eCB mobilization, whereby a 1 Hz in vivo-like sparse spike train that was similar to the spike firing rate observed during learning and memory in vivo [49, 50] was combined with DHPG application, which activated group 1 mGluR (Figs. 1, 2). As depolarization of hippocampal CA1 PCs [19, 69] and activation of group 1 mGluR [31] are likely to occur concomitantly in the hippocampus during memory processing in vivo [70, 71]*,* it may be speculated that PLCβ-dependent S-eCB mobilization may be predominant under in vivo conditions. Moreover, our novel S-eCB mobilization paradigm induced changes in eIPSCs that were similar to those observed when using physiologically unrealistic strong and prolonged postsynaptic depolarization [29, 32, 36], underscoring the importance of physiologically realistic S-eCB mobilization used in this study. Notably, using our novel S-eCB mobilization paradigm, we demonstrated, for the first time, that AβO completely impairs PLCβ-dependent S-eCB mobilization in the hippocampus (Figs. 1, 2) and, more importantly, that it can be restored by the application of the PLC activator, m-3M3FBS. This was possible because AβO significantly reduced hippocampal PLCβ1 protein levels in AβO-treated rat hippocampal slices (Fig. 2), which could also be reinstated by the application of m-3M3FBS, as confirmed by western blot analysis. Therefore, our results demonstrate the necessity and sufficiency of the involvement of the PLCβ-dependent pathway in AβO-mediated impairment of S-eCB mobilization. Although the disruption of PLCβ1-mediated signaling in the brain is associated with epilepsy, schizophrenia, and bipolar disorder [72–74], to the best of our knowledge, our results provide the first direct evidence of the disruption of the hippocampal PLCβ1 pathway in a rodent model of AD.

It is already well established that eCB mobilization is important for hippocampal LTP at excitatory synapses [14, 15]. Prolonged postsynaptic depolarization before LTP induction facilitated LTP induction by enhancing [Ca^2+^]_i_-dependent eCB mobilization [14]. In addition, blocking the receptors of eCB by applying the CB1R receptor antagonist, AM251, blocked LTP induction [15]. However, the effect of PLCβ-dependent eCB or S-eCB mobilization on hippocampal LTP induction has not been investigated. Here, using our novel PLCβ-dependent S-eCB mobilization paradigm, we demonstrated that PLCβ-dependent S-eCB mobilization can induce NMDAR-dependent tLTP at hippocampal CA3-CA1 synapses (Fig. 3c–e), or even convert mGluR5-mediated LTD to NMDAR-dependent tLTP (Fig. 3c). Since our unique PLCβ-dependent S-eCB mobilization protocol led to the reduction of eIPSC amplitudes in CA1 PC somata, disinhibition may have contributed to the facilitation of hippocampal tLTP induction at CA3-CA1 excitatory synapses by facilitating the back-propagating spikes in the dendrites of CA1 PC [75, 76], which are required for the induction of tLTP. However, as tLTP induction was completely blocked by AβO in our study (Fig. 3f), AβO may have disrupted the PLCβ1 pathway to disrupt CB1R-dependent S-eCB mobilization. CB1R activation modulates excitatory [77–79] and inhibitory hippocampal synaptic transmissions [29, 30, 36, 69, 80, 81], both of which are known to be disrupted by AβO via CB1R dysfunction [28, 41, 82]. Thus, it is possible that AβO may have broken the fine balance between the excitation/inhibition (E/I) required for hippocampal LTP induction [11, 83]. In fact, AβO has been shown to inhibit E-S potentiation by disrupting CB1R-mediated DSI in the rat hippocampal slices [41]. In our study, the AβO-mediated impairment of tLTP induction was fully restored by m-3M3FBS (Fig. 3g). This suggests the possibility that m-3M3FBS may have rescued the E/I balance by enhancing eCB mobilization in AβO-treated rat hippocampal slices, leading to the recovery of tLTP induction. Although this was not characterized in our study, it would be interesting to directly test this hypothesis in the future. In addition to the contributions of AβO-mediated disruption of E/I balance to tLTP impairment, AβO may have caused dysfunctions of the mGluR5 receptor itself [84, 85], which could have led to the elevation of glutamate spillover at hippocampal excitatory synapses [11, 86]. However, it is likely that S-eCB mobilization, E/I balance, and mGluR5 all contributed in parts to the AβO-mediated impairments of S-eCB mobilization-induced tLTP.

Our results are the first to show that increasing PLCβ1 activity via m-3M3FBS application rescues hippocampal tLTP induction (Fig. 3) and contextual fear memory (Fig. 4) impaired by amyloidosis, possibly by restoring S-eCB mobilization (Fig. 2). In fact, some studies reported that the levels of PLCβ1 protein in the hippocampus rise immediately after CFC [87] or auditory fear conditioning [88]. Moreover, the infusion of m-3M3FBS in the basolateral amygdala increased memory consolidation after CFC [89]. In fact, the m-3M3FBS used in our study is commonly used to activate, not only PLCβ in vitro [90], but also postsynaptic upstream signaling of PLCβ-dependent eCB signaling, such as DAG production [91] or increases in [Ca^2+^]_i_ [92]. Thus, it would be interesting to test whether DAG production or [Ca^2+^]_i_ itself contributes to the restoration of tLTP and contextual fear memory impairment. Methodologically, although we directly injected m-3M3FBS into the dorsal and ventral hippocampi of 5XFAD mice, other studies administered m-3M3FBS via intraperitoneal injections [93, 94]. To enhance the therapeutic effectiveness in neurological disorders, it remains to be determined whether oral administration or intraperitoneal injection of m-3M3FBS can pass the blood-brain barrier to activate PLCβ directly.

However, in contrast to our results, some studies have reported that postsynaptic mGluR5 acts as a receptor for AβO [84, 95, 96]. Other studies have reported that mGluR5 antagonists prevent the pathologic effect of AβO by suppressing the interaction between cellular prion protein (PrP^C^) and mGluR5 signaling. The discrepancy between these results may be due to the varied targets of the mGluR5 pathways. In our study, we targeted the mGluR5-PLCβ-DAG-eCB pathway [17] to determine the pathological mechanism of AβO, while other studies targeted the Homer1-calcium/calmodulin-dependent eukaryotic elongation factor 2 kinase [84, 97–100] from the PrP^C^-mGluR5 complex. In fact, activation of mGluR5 in our study induced mGluR-mediated LTD in the control pathways (Fig. 3c), similar to other studies [61, 101] and such mGluR5-mediated LTD was unaffected by AβO (Fig. 3f). These results indicate that mGluR5-mediated LTD is robust to AβO [102] and is independent of mGluR5-mediated PLCβ-dependent eCB mobilization, which is consistent with previous studies [103, 104]. Therefore, it is crucial to target more specific downstream pathways of mGluR5 to fully understand the effect of AβO on mGluR5.

Overall, our results demonstrate, for the first time, that AβO reduces PLCβ1 protein levels to disrupt S-eCB mobilization, which, in turn, impairs tLTP and hippocampus-dependent contextual fear memory. These results not only underscore the critical role of PLCβ-dependent S-eCB mobilization in hippocampal synaptic plasticity and memory in the healthy brains, but also suggest that PLCβ1 could serve as a therapeutic target for improving hippocampal synaptic plasticity and memory-impaired by amyloidosis in AD.

## Conclusion

Taken together, our results suggest that enhancing eCB mobilization by increasing PLCβ1 protein levels could have therapeutic potential for restoring memory impairments in both early and chronic stages of AD.

### Limitations

This study had several limitations. First, different species of rodents were used in this study. We used SD rats for all in vitro studies, while mice were used for all contextual fear memory experiments in vivo. Differences in hippocampal electrophysiological features between rat and mouse species are already well established [105], and as all of the in vitro studies were performed using SD rats, it would have been appropriate to use an SD rat model of AD in testing contextual fear memory experiments. In fact, transgenic SD rat models of AD that can mimic extracellular Aβ deposition following the amyloid hypothesis exist [44, 45]. However, while memory impairments were observed at 9 months in this rat model of AD, hippocampal Aβ deposition was observed at 17–18 months [45, 106]. As memory impairments precede Aβ deposition, they do not follow the amyloid hypothesis of AD [3]. As our study was intended to investigate the effect of Aβ deposition on PLCβ1 protein levels and hippocampus-dependent contextual fear memory, we chose to use the 5XFAD mice that display Aβ deposition from 2 months, which precedes the onset of contextual fear memory deficit that emerges from 6 to 7 months [42, 68]. Thus, to directly investigate the effect of Aβ deposition, we used 5XFAD mice for the contextual fear memory test, despite being of different species compared to the SD rat model used for the investigation of the acute effect of AβO on PLCβ.

Second, we performed in vitro electrophysiology using SD rats at 2 to 3 weeks postnatal, while in vivo hippocampus-dependent contextual fear memory was tested using 6- to 7-month-old adult C57BL/6 or 5XFAD mice. It is possible that these age differences may have caused significant differences in physiological functions, including mGluR function [107] and eCB mobilization [39, 108] as they both mature with development; thus, S-eCB mobilization should be tested in 6- to 7-month-old adult C57BL/6 or 5XFAD mice in the future.

Finally, we used C57BL/6 mice as WT controls for the experimental 5XFAD mice group, but littermates that were generated by crossing hemizygous 5XFAD mice should have been used as WT controls for 5XFAD mice.

## Data Availability

The datasets used and/or analyzed during the current study are available from the corresponding author upon request.

## Abbreviations

ACSF: Artificial cerebrospinal fluid
AβO: Amyloid beta oligomers
AD: Alzheimer’s disease
ANOVA: Analysis of variance
Ca^2+^: Calcium
CA1: Cornus Ammonis1
CA3: Cornus Ammonis3
CB1R: Cannabinoid type 1 receptor
CFC: Contextual fear conditioning
DAG: Diacylglycerol
DMSO: Dimethyl sulfoxide
DNA: *Deoxyribonucleic acid*
DSI: Depolarization-induced suppression of inhibition
eCB: Endocannabinoid
eIPSC: Evoked inhibitory postsynaptic current
EPSP: Excitatory postsynaptic potential
E/I: Excitation/inhibition balance
E-S: EPSP-spike
GABA: Gamma-aminobutyric acid
GAPDH: Glyceraldehyde-3-phosphate dehydrogenase
LTD: Long-term depression
LTP: Long-term potentiation
MAGL: Monoacylglycerol
mGluR: Metabotrophic glutamate receptor
NMDAR: *N*-methyle-D-aspartate receptor
PC: Pyramidal cell
PCR: Polymerase chain reaction
PLCβ: Phospholipase Cβ
PrP^C^: Cellular prion protein
SC: Schaffer collateral
SD: Sprague-Dawley
S-eCB: Synergistic enhancement of endocannabinoid
SEM: Standard error of the mean
tLTP: Spike timing-dependent potentiation
WT: Wild type
2-AG: 2-arachidonoyglycerol

## References

[1] Price DL (1986). New perspectives on Alzheimer's disease. Annu Rev Neurosci.

[2] Selkoe DJ (2000). Toward a comprehensive theory for Alzheimer's disease. Hypothesis: Alzheimer's disease is caused by the cerebral accumulation and cytotoxicity of amyloid beta-protein. Ann N Y Acad Sci.

[3] Hardy J, Selkoe DJ (2002). The amyloid hypothesis of Alzheimer's disease: progress and problems on the road to therapeutics. Science.

[4] Lambert MP, Barlow AK, Chromy BA, Edwards C, Freed R, Liosatos M, Morgan TE, Rozovsky I, Trommer B, Viola KL, Wals P, Zhang C, Finch CE, Krafft GA, Klein WL (1998). Diffusible, nonfibrillar ligands derived from Abeta1-42 are potent central nervous system neurotoxins. Proc Natl Acad Sci U S A.

[5] McLean CA, Cherny RA, Fraser FW, Fuller SJ, Smith MJ, Beyreuther K, Bush AI, Masters CL (1999). Soluble pool of Abeta amyloid as a determinant of severity of neurodegeneration in Alzheimer's disease. Ann Neurol.

[6] Lacor PN, Buniel MC, Chang L, Fernandez SJ, Gong Y, Viola KL, Lambert MP, Velasco PT, Bigio EH, Finch CE, Krafft GA, Klein WL (2004). Synaptic targeting by Alzheimer's-related amyloid beta oligomers. J Neurosci.

[7] Shankar GM, Li S, Mehta TH, Garcia-Munoz A, Shepardson NE, Smith I, Brett FM, Farrell MA, Rowan MJ, Lemere CA, Regan CM, Walsh DM, Sabatini BL, Selkoe DJ (2008). Amyloid-beta protein dimers isolated directly from Alzheimer's brains impair synaptic plasticity and memory. Nat Med.

[8] Malaplate-Armand C, Florent-Bechard S, Youssef I, Koziel V, Sponne I, Kriem B, Leininger-Muller B, Olivier JL, Oster T, Pillot T (2006). Soluble oligomers of amyloid-beta peptide induce neuronal apoptosis by activating a cPLA2-dependent sphingomyelinase-ceramide pathway. Neurobiol Dis.

[9] Alberdi E, Sanchez-Gomez MV, Cavaliere F, Perez-Samartin A, Zugaza JL, Trullas R, Domercq M, Matute C (2010). Amyloid beta oligomers induce Ca2+ dysregulation and neuronal death through activation of ionotropic glutamate receptors. Cell Calcium.

[10] Wang HW, Pasternak JF, Kuo H, Ristic H, Lambert MP, Chromy B, Viola KL, Klein WL, Stine WB, Krafft GA, Trommer BL (2002). Soluble oligomers of beta amyloid (1-42) inhibit long-term potentiation but not long-term depression in rat dentate gyrus. Brain Res.

[11] Lei M, Xu H, Li Z, Wang Z, O'Malley TT, Zhang D, Walsh DM, Xu P, Selkoe DJ, Li S (2016). Soluble Abeta oligomers impair hippocampal LTP by disrupting glutamatergic/GABAergic balance. Neurobiol Dis.

[12] Neves G, Cooke SF, Bliss TV (2008). Synaptic plasticity, memory and the hippocampus: a neural network approach to causality. Nat Rev Neurosci.

[13] Martin SJ, Grimwood PD, Morris RG (2000). Synaptic plasticity and memory: an evaluation of the hypothesis. Ann Rev Neurosci.

[14] Carlson G, Wang Y, Alger BE (2002). Endocannabinoids facilitate the induction of LTP in the hippocampus. Nat Neurosci.

[15] Chevaleyre V, Castillo PE (2004). Endocannabinoid-mediated metaplasticity in the hippocampus. Neuron.

[16] Isokawa M, Alger BE (2006). Ryanodine receptor regulates endogenous cannabinoid mobilization in the hippocampus. J Neurophysiol.

[17] Kano M, Ohno-Shosaku T, Hashimotodani Y, Uchigashima M, Watanabe M (2009). Endocannabinoid-mediated control of synaptic transmission. Physiol Rev.

[18] Alger BE (2002). Retrograde signaling in the regulation of synaptic transmission: focus on endocannabinoids. Prog Neurobiol.

[19] Pitler TA, Alger BE (1994). Depolarization-induced suppression of GABAergic inhibition in rat hippocampal pyramidal cells: G protein involvement in a presynaptic mechanism. Neuron.

[20] Katona I, Sperlagh B, Sik A, Kafalvi A, Vizi ES, Mackie K, Freund TF (1999). Presynaptically located CB1 cannabinoid receptors regulate GABA release from axon terminals of specific hippocampal interneurons. J Neurosci.

[21] Zhu PJ, Lovinger DM (2007). Persistent synaptic activity produces long-lasting enhancement of endocannabinoid modulation and alters long-term synaptic plasticity. J Neurophysiol.

[22] Xu JY, Zhang J, Chen C (2012). Long-lasting potentiation of hippocampal synaptic transmission by direct cortical input is mediated via endocannabinoids. J Physiol.

[23] Ramirez BG, Blazquez C, Gomez del Pulgar T, Guzman M, de Ceballos ML (2005). Prevention of Alzheimer's disease pathology by cannabinoids: neuroprotection mediated by blockade of microglial activation. J Neurosci.

[24] Kalifa S, Polston EK, Allard JS, Manaye KF (2011). Distribution patterns of cannabinoid CB1 receptors in the hippocampus of APPswe/PS1DeltaE9 double transgenic mice. Brain Res.

[25] Aso E, Palomer E, Juves S, Maldonado R, Munoz FJ, Ferrer I (2012). CB1 agonist ACEA protects neurons and reduces the cognitive impairment of AbetaPP/PS1 mice. J Alzheimers Dis.

[26] Haghani M, Shabani M, Javan M, Motamedi F, Janahmadi M (2012). CB1 cannabinoid receptor activation rescues amyloid beta-induced alterations in behaviour and intrinsic electrophysiological properties of rat hippocampal CA1 pyramidal neurones. Cell Physiol Biochem.

[27] Cheng D, Spiro AS, Jenner AM, Garner B, Karl T (2014). Long-term cannabidiol treatment prevents the development of social recognition memory deficits in Alzheimer's disease transgenic mice. J Alzheimers Dis.

[28] Chen R, Zhang J, Wu Y, Wang D, Feng G, Tang YP, Teng Z, Chen C (2012). Monoacylglycerol lipase is a therapeutic target for Alzheimer's disease. Cell Rep.

[29] Wilson RI, Nicoll RA (2001). Endogenous cannabinoids mediate retrograde signalling at hippocampal synapses. Nature.

[30] Varma N, Carlson GC, Ledent C, Alger BE (2001). Metabotropic glutamate receptors drive the endocannabinoid system in hippocampus. J Neurosci.

[31] Morishita W, Kirov SA, Alger BE (1998). Evidence for metabotropic glutamate receptor activation in the induction of depolarization-induced suppression of inhibition in hippocampal CA1. J Neurosci.

[32] Ohno-Shosaku T, Maejima T, Kano M (2001). Endogenous cannabinoids mediate retrograde signals from depolarized postsynaptic neurons to presynaptic terminals. Neuron.

[33] Hashimotodani Y, Ohno-Shosaku T, Tsubokawa H, Ogata H, Emoto K, Maejima T, Araishi K, Shin HS, Kano M (2005). Phospholipase Cbeta serves as a coincidence detector through its Ca2+ dependency for triggering retrograde endocannabinoid signal. Neuron.

[34] Rimmerman N, Hughes HV, Bradshaw HB, Pazos MX, Mackie K, Prieto AL, Walker JM (2008). Compartmentalization of endocannabinoids into lipid rafts in a dorsal root ganglion cell line. Br J Pharmacol.

[35] Di Marzo V, Melck D, Bisogno T, De Petrocellis L (1998). Endocannabinoids: endogenous cannabinoid receptor ligands with neuromodulatory action. Trends Neurosci.

[36] Ohno-Shosaku T, Shosaku J, Tsubokawa H, Kano M (2002). Cooperative endocannabinoid production by neuronal depolarization and group I metabotropic glutamate receptor activation. Eur J Neurosci.

[37] Tanimura A, Yamazaki M, Hashimotodani Y, Uchigashima M, Kawata S, Abe M, Kita Y, Hashimoto K, Shimizu T, Watanabe M, Sakimura K, Kano M (2010). The endocannabinoid 2-arachidonoylglycerol produced by diacylglycerol lipase alpha mediates retrograde suppression of synaptic transmission. Neuron.

[38] Gao Y, Vasilyev DV, Goncalves MB, Howell FV, Hobbs C, Reisenberg M, Shen R, Zhang MY, Strassle BW, Lu P, Mark L, Piesla MJ, Deng K, Kouranova EV, Ring RH, Whiteside GT, Bates B, Walsh FS, Williams G, Pangalos MN, Samad TA, Doherty P (2010). Loss of retrograde endocannabinoid signaling and reduced adult neurogenesis in diacylglycerol lipase knock-out mice. J Neurosci.

[39] Liang SL, Alger BE, McCarthy MM (2014). Developmental increase in hippocampal endocannabinoid mobilization: role of metabotropic glutamate receptor subtype 5 and phospholipase C. J Neurophysiol.

[40] Mulder J, Zilberter M, Pasquare SJ, Alpar A, Schulte G, Ferreira SG, Kofalvi A, Martin-Moreno AM, Keimpema E, Tanila H (2011). Molecular reorganization of endocannabinoid signalling in Alzheimer's disease. Brain.

[41] Orr AL, Hanson JE, Li D, Klotz A, Wright S, Schenk D, Seubert P, Madison DV (2014). beta-Amyloid inhibits E-S potentiation through suppression of cannabinoid receptor 1-dependent synaptic disinhibition. Neuron.

[42] Oakley H, Cole SL, Logan S, Maus E, Shao P, Craft J, Guillozet-Bongaarts A, Ohno M, Disterhoft J, Van Eldik L (2006). Intraneuronal beta-amyloid aggregates, neurodegeneration, and neuron loss in transgenic mice with five familial Alzheimer's disease mutations: potential factors in amyloid plaque formation. J Neurosci.

[43] Benedikz E, Kloskowska E, Winblad B (2009). The rat as an animal model of Alzheimer's disease. J Cell Mol Med.

[44] Folkesson R, Malkiewicz K, Kloskowska E, Nilsson T, Popova E, Bogdanovic N, Ganten U, Ganten D, Bader M, Winblad B, Benedikz E (2007). A transgenic rat expressing human APP with the Swedish Alzheimer's disease mutation. Biochem Biophys Res Commun.

[45] Flood DG, Lin YG, Lang DM, Trusko SP, Hirsch JD, Savage MJ, Scott RW, Howland DS (2009). A transgenic rat model of Alzheimer's disease with extracellular Abeta deposition. Neurobiol Aging.

[46] Park K, Lee J, Jang HJ, Richards BA, Kohl MM, Kwag J (2020). Optogenetic activation of parvalbumin and somatostatin interneurons selectively restores theta-nested gamma oscillations and oscillation-induced spike timing-dependent long-term potentiation impaired by amyloid beta oligomers. BMC Biol.

[47] Vadukul DM, Gbajumo O, Marshall KE, Serpell LC (2017). Amyloidogenicity and toxicity of the reverse and scrambled variants of amyloid-beta 1-42. FEBS Lett.

[48] Alger BE, Pitler TA, Wagner JJ, Martin LA, Morishita W, Kirov SA, Lenz RA (1996). Retrograde signalling in depolarization-induced suppression of inhibition in rat hippocampal CA1 cells. J Physiol.

[49] Mizuseki K, Buzsaki G (2013). Preconfigured, skewed distribution of firing rates in the hippocampus and entorhinal cortex. Cell Rep.

[50] Csicsvari J, Hirase H, Czurko A, Mamiya A, Buzsaki G (1999). Oscillatory coupling of hippocampal pyramidal cells and interneurons in the behaving Rat. J Neurosci.

[51] Kwag J, Paulsen O (2012). Gating of NMDA receptor-mediated hippocampal spike timing-dependent potentiation by mGluR5. Neuropharmacology.

[52] Mahmood T, Yang PC (2012). Western blot: technique, theory, and trouble shooting. N Am J Med Sci.

[53] Laemmli UK (1970). Cleavage of structural proteins during the assembly of the head of bacteriophage T4. Nature.

[54] Paxinos G, Franklin KBJ. Paxinos and Franklin's the mouse brain in stereotaxic coordinates. 5th Ed. San Diego: Elsevier Academic Press; 2019.

[55] Pham J, Cabrera SM, Sanchis-Segura C, Wood MA (2009). Automated scoring of fear-related behavior using EthoVision software. J Neurosci Methods.

[56] Chevaleyre V, Castillo PE (2003). Heterosynaptic LTD of hippocampal GABAergic synapses: a novel role of endocannabinoids in regulating excitability. Neuron.

[57] Nakanishi S (1992). Molecular diversity of glutamate receptors and implications for brain function. Science.

[58] Conn PJ, Pin JP (1997). Pharmacology and functions of metabotropic glutamate receptors. Annu Rev Pharmacol Toxicol.

[59] Watanabe M, Nakamura M, Sato K, Kano M, Simon MI, Inoue Y (1998). Patterns of expression for the mRNA corresponding to the four isoforms of phospholipase Cbeta in mouse brain. Eur J Neurosci.

[60] Bolshakov VY, Siegelbaum SA (1994). Postsynaptic induction and presynaptic expression of hippocampal long-term depression. Science.

[61] Palmer MJ, Irving AJ, Seabrook GR, Jane DE, Collingridge GL (1997). The group I mGlu receptor agonist DHPG induces a novel form of LTD in the CA1 region of the hippocampus. Neuropharmacology.

[62] Markram H, Gerstner W, Sjostrom PJ (2011). A history of spike-timing-dependent plasticity. Front Synaptic Neurosci.

[63] Markram H, Lubke J, Frotscher M, Sakmann B (1997). Regulation of synaptic efficacy by coincidence of postsynaptic APs and EPSPs. Science.

[64] Bi G, Poo M (2001). Synaptic modification by correlated activity: Hebb's postulate revisited. Annu Rev Neurosci.

[65] Phillips RG, LeDoux JE (1992). Differential contribution of amygdala and hippocampus to cued and contextual fear conditioning. Behav Neurosci.

[66] Holland PC, Bouton ME (1999). Hippocampus and context in classical conditioning. Curr Opin Neurobiol.

[67] Kimura R, Ohno M (2009). Impairments in remote memory stabilization precede hippocampal synaptic and cognitive failures in 5XFAD Alzheimer mouse model. Neurobiol Dis.

[68] Ohno M (2009). Failures to reconsolidate memory in a mouse model of Alzheimer's disease. Neurobiol Learn Mem.

[69] Pitler TA, Alger BE (1992). Postsynaptic spike firing reduces synaptic GABAA responses in hippocampal pyramidal cells. J Neurosci.

[70] Naie K, Manahan-Vaughan D (2004). Regulation by metabotropic glutamate receptor 5 of LTP in the dentate gyrus of freely moving rats: relevance for learning and memory formation. Cereb Cortex.

[71] Mukherjee S, Manahan-Vaughan D (2013). Role of metabotropic glutamate receptors in persistent forms of hippocampal plasticity and learning. Neuropharmacology.

[72] Kurian MA, Meyer E, Vassallo G, Morgan NV, Prakash N, Pasha S, Hai NA, Shuib S, Rahman F, Wassmer E, Cross JH, O’Callaghan FJ, Osborne JP, Scheffer IE, Gissen P, Maher ER (2010). Phospholipase C beta 1 deficiency is associated with early-onset epileptic encephalopathy. Brain.

[73] Lo Vasco VR, Longo L, Polonia P (2013). Phosphoinositide-specific phospholipase C beta1 gene deletion in bipolar disorder affected patient. J Cell Commun Signal.

[74] Poduri A, Chopra SS, Neilan EG, Elhosary PC, Kurian MA, Meyer E, Barry BJ, Khwaja OS, Salih MA, Stodberg T (2012). Homozygous PLCB1 deletion associated with malignant migrating partial seizures in infancy. Epilepsia.

[75] Spruston N, Schiller Y, Stuart G, Sakmann B (1995). Activity-dependent action potential invasion and calcium influx into hippocampal CA1 dendrites. Science.

[76] Magee JC, Johnston D (1997). A synaptically controlled, associative signal for Hebbian plasticity in hippocampal neurons. Science.

[77] Ohno-Shosaku T, Tsubokawa H, Mizushima I, Yoneda N, Zimmer A, Kano M (2002). Presynaptic cannabinoid sensitivity is a major determinant of depolarization-induced retrograde suppression at hippocampal synapses. J Neurosci.

[78] Shen M, Piser TM, Seybold VS, Thayer SA (1996). Cannabinoid receptor agonists inhibit glutamatergic synaptic transmission in rat hippocampal cultures. J Neurosci.

[79] Misner DL, Sullivan JM (1999). Mechanism of cannabinoid effects on long-term potentiation and depression in hippocampal CA1 neurons. J Neurosci.

[80] Hoffman AF, Lupica CR (2000). Mechanisms of cannabinoid inhibition of GABA(A) synaptic transmission in the hippocampus. J Neurosci.

[81] Szabo B, Dorner L, Pfreundtner C, Norenberg W, Starke K (1998). Inhibition of GABAergic inhibitory postsynaptic currents by cannabinoids in rat corpus striatum. Neuroscience.

[82] Maccarrone M, Totaro A, Leuti A, Giacovazzo G, Scipioni L, Mango D, Coccurello R, Nistico R, Oddi S (2018). Early alteration of distribution and activity of hippocampal type-1 cannabinoid receptor in Alzheimer's disease-like mice overexpressing the human mutant amyloid precursor protein. Pharmacol Res.

[83] Hashimotodani Y, Nasrallah K, Jensen KR, Chavez AE, Carrera D, Castillo PE (2017). LTP at Hilar mossy cell-dentate granule cell synapses modulates dentate gyrus output by increasing excitation/inhibition balance. Neuron.

[84] Haas LT, Strittmatter SM (2016). Oligomers of amyloid beta prevent physiological activation of the cellular prion protein-metabotropic glutamate receptor 5 complex by glutamate in Alzheimer disease. J Biol Chem.

[85] Hamilton A, Zamponi GW, Ferguson SS (2015). Glutamate receptors function as scaffolds for the regulation of beta-amyloid and cellular prion protein signaling complexes. Mol Brain.

[86] Li S, Hong S, Shepardson NE, Walsh DM, Shankar GM, Selkoe D (2009). Soluble oligomers of amyloid Beta protein facilitate hippocampal long-term depression by disrupting neuronal glutamate uptake. Neuron.

[87] Weeber EJ, Savage DD, Sutherland RJ, Caldwell KK (2001). Fear conditioning-induced alterations of phospholipase C-beta1a protein level and enzyme activity in rat hippocampal formation and medial frontal cortex. Neurobiol Learn Mem.

[88] Buckley CT, Caldwell KK (2004). Fear conditioning is associated with altered integration of PLC and ERK signaling in the hippocampus. Pharmacol Biochem Behav.

[89] Ouyang M, Young MB, Lestini MM, Schutsky K, Thomas SA (2012). Redundant catecholamine signaling consolidates fear memory via phospholipase C. J Neurosci.

[90] Bae YS, Lee TG, Park JC, Hur JH, Kim Y, Heo K, Kwak JY, Suh PG, Ryu SH (2003). Identification of a compound that directly stimulates phospholipase C activity. Mol Pharmacol.

[91] Myeong J, Ko J, Kwak M, Kim J, Woo J, Ha K, Hong C, Yang D, Kim HJ, Jeon JH, So I (2018). Dual action of the Galphaq-PLCbeta-PI(4,5)P2 pathway on TRPC1/4 and TRPC1/5 heterotetramers. Sci Rep.

[92] Szebenyi SA, Ogura T, Sathyanesan A, AlMatrouk AK, Chang J, Lin W (2014). Increases in intracellular calcium via activation of potentially multiple phospholipase C isozymes in mouse olfactory neurons. Front Cell Neurosci.

[93] Wang L, Zhou Y, Chen Z, Sun L, Wu J, Li H, Liu F, Wang F, Yang C, Yang J, Leng Q, Zhang Q, Xu A, Shen L, Sun J, Wu D, Fang C, Lu H, Yan D, Ge B (2019). PLCbeta2 negatively regulates the inflammatory response to virus infection by inhibiting phosphoinositide-mediated activation of TAK1. Nat Commun.

[94] Kim SD, Kim HJ, Shim JW, Lee HY, Lee SK, Kwon S, Jung YS, Baek SH, Park JS, Zabel BA, Bae YS (2012). Phospholipase C activator m-3M3FBS protects against morbidity and mortality associated with sepsis. J Immunol.

[95] Renner M, Lacor PN, Velasco PT, Xu J, Contractor A, Klein WL, Triller A (2010). Deleterious effects of amyloid beta oligomers acting as an extracellular scaffold for mGluR5. Neuron.

[96] Um JW, Kaufman AC, Kostylev M, Heiss JK, Stagi M, Takahashi H, Kerrisk ME, Vortmeyer A, Wisniewski T, Koleske AJ, Gunther EC, Nygaard HB, Strittmatter SM (2013). Metabotropic glutamate receptor 5 is a coreceptor for Alzheimer abeta oligomer bound to cellular prion protein. Neuron.

[97] Brakeman PR, Lanahan AA, O'Brien R, Roche K, Barnes CA, Huganir RL, Worley PF (1997). Homer: a protein that selectively binds metabotropic glutamate receptors. Nature.

[98] Mao L, Yang L, Tang Q, Samdani S, Zhang G, Wang JQ (2005). The scaffold protein Homer1b/c links metabotropic glutamate receptor 5 to extracellular signal-regulated protein kinase cascades in neurons. J Neurosci.

[99] Park S, Park JM, Kim S, Kim JA, Shepherd JD, Smith-Hicks CL, Chowdhury S, Kaufmann W, Kuhl D, Ryazanov AG, Huganir RL, Linden DJ, Worley PF (2008). Elongation factor 2 and fragile X mental retardation protein control the dynamic translation of Arc/Arg3.1 essential for mGluR-LTD. Neuron.

[100] Haas LT, Salazar SV, Kostylev MA, Um JW, Kaufman AC, Strittmatter SM (2016). Metabotropic glutamate receptor 5 couples cellular prion protein to intracellular signalling in Alzheimer's disease. Brain.

[101] Huber KM, Roder JC, Bear MF (2001). Chemical induction of mGluR5- and protein synthesis--dependent long-term depression in hippocampal area CA1. J Neurophysiol.

[102] Yang W, Zhou X, Zimmermann HR, Cavener DR, Klann E, Ma T (2016). Repression of the eIF2alpha kinase PERK alleviates mGluR-LTD impairments in a mouse model of Alzheimer's disease. Neurobiol Aging.

[103] Kim HH, Park JM, Lee SH, Ho WK (2019). Association of mGluR-dependent LTD of excitatory synapses with endocannabinoid-dependent LTD of inhibitory synapses leads to EPSP to spike potentiation in CA1 pyramidal neurons. J Neurosci.

[104] Kauer JA, Malenka RC (2007). Synaptic plasticity and addiction. Nat Rev Neurosci.

[105] Routh BN, Johnston D, Harris K, Chitwood RA (2009). Anatomical and electrophysiological comparison of CA1 pyramidal neurons of the rat and mouse. J Neurophysiol.

[106] Kloskowska E, Pham TM, Nilsson T, Zhu S, Oberg J, Codita A, Pedersen LA, Pedersen JT, Malkiewicz K, Winblad B, Folkesson R, Benedikz E (2010). Cognitive impairment in the Tg6590 transgenic rat model of Alzheimer's disease. J Cell Mol Med.

[107] Nosyreva ED, Huber KM (2005). Developmental switch in synaptic mechanisms of hippocampal metabotropic glutamate receptor-dependent long-term depression. J Neurosci.

[108] Piyanova A, Lomazzo E, Bindila L, Lerner R, Albayram O, Ruhl T, Lutz B, Zimmer A, Bilkei-Gorzo A (2015). Age-related changes in the endocannabinoid system in the mouse hippocampus. Mech Ageing Dev.

